# Fine Mapping, Candidate Gene Identification and Co-segregating Marker Development for the Phytophthora Root Rot Resistance Gene *RpsYD25*

**DOI:** 10.3389/fgene.2020.00799

**Published:** 2020-07-28

**Authors:** Chao Zhong, Suli Sun, Xuecui Zhang, Canxing Duan, Zhendong Zhu

**Affiliations:** ^1^Institute of Crop Sciences, Chinese Academy of Agricultural Sciences, Beijing, China; ^2^College of Agronomy, Shenyang Agricultural University, Shenyang, China

**Keywords:** Phytophthora root rot, fine mapping, resistance gene, *RpsYD25*, soybean

## Abstract

Phytophthora root rot (PRR) caused by *Phytophthora sojae* is a serious disease of soybean. The most effective disease-control strategy is to deploy resistant cultivars carrying *Rps* genes. Soybean cultivar Yudou25 can effectively resist pathotypes of *P. sojae* in China. Previous studies have mapped the *Rps* gene in Yudou25, *RpsYD25*, on chromosome 3. In this study, at first *RpsYD25* was located between SSR markers Satt1k3 (2.2 cM) and BARCSOYSSR_03_0253 (4.5 cM) by using an F_2:3_ population containing 165 families derived from Zaoshu18 and Yudou25. Then the recombination sites were identified in 1127 F_3:4_ families derived from Zaoshu18 and Yudou25 using the developed PCR-based SNP, InDel and SSR markers, and *RpsYD25* was finely mapped in the a 101.3 kb genomic region. In this region, a zinc ion binding and nucleic acid binding gene Glyma.03g034700 and two NBS-LRR genes Glyma.03g034800 and Glyma.03g034900 were predicted as candidate genes of *RpsYD25*, and five co-segregated SSR markers with *RpsYD25* were identified and validated to be diagnostic markers. Combined with the resistance reaction to multiple *P. sojae* isolates, seven of 178 soybean genotypes were detected to contain *RpsYD25* using the five co-segregated SSR markers. The soybean genotypes carrying *RpsYD25* and the developed co-segregated markers can be effectively applied in the breeding for *P. sojae* resistance in China.

## Introduction

Phytophthora root rot (PRR), caused by the soil-borne oomycete pathogen *Phytophthora sojae*, is one of the most devastative diseases in soybean-growing regions worldwide, since it was firstly reported in Indiana, United States (Kaufmann and Gerdemann, 1958; Schmitthenner, 1985; Kamoun et al., 2015). According to the latest reports, from 2006 to 2014, approximately 340 million bushels of yield losses were attributed to PRR in major soybean production areas of Unite States, and PRR causes economic losses of $1 – 2 billion annually worldwide (Tyler, 2007; Koenning and Wrather, 2010). The disease was first reported in Northeast China and has now spread to all major soybean producing areas in China (Zhu et al., 2003; Tian et al., 2016; Wu et al., 2016). Under saturated soil conditions, *P. sojae* infect soybean plants throughout the growing season, resulting in pre- and post-emergence damping-off, root and stem rot, yellowing and wilting of leaves, and the death of soybean plants (Schmitthenner, 1999; Chen and Wang, 2017). At present, the most economical and eco-friendly method to control PRR is deployment of resistant soybean cultivars (Kamoun et al., 2015; Dorrance, 2018).

Two different types of PRR resistance have been identified in soybean, complete resistance and partial resistance (Sugimoto et al., 2012). Complete resistance is race-specific and monogenic, and is the result of a single dominant resistance *Rps* gene that confers immunity to *P. sojae*. In contrast, partial resistance appears as quantitative trait locus (QTL) loci controlled by multiple genes (Tooley and Grau, 1984; Dorrance et al., 2003b, 2004; Sugimoto et al., 2012). Since qualitative resistance is controlled by a single gene, it is easier to introduce into susceptible cultivars, and facilitates the selection of phenotypes during breeding, discovery of utilization of the *Rps* genes has always been the focus of research. Up to now, nearly 30 *Rps* genes have been identified and mapped to nine chromosomes, which are distributed on chromosomes 2, 3, 7, 10, 13, and 16–19 (Sugimoto et al., 2012; Lin et al., 2013; Zhang et al., 2013a, b; Sun et al., 2014; Li et al., 2016, 2017; Ping et al., 2016; Cheng et al., 2017; Niu et al., 2017; Sahoo et al., 2017; Zhong et al., 2017, 2018). Among the nine chromosomes containing *Rps* genes, chromosome 3 has much more *Rps* genes identified than other chromosomes. So far, 15 identified *Rps* genes are known to be distributed on the short arm of chromosome 3 in soybean, they were *Rps1* alleles, *Rps7*, *RpsYD25*, *RpsYD29*, *Rps9*, *RpsQ*, *RpsUN1*, the *Rps* gene in Waseshiroge, *RpsWY*, *RpsHN*, and *RpsHC1*8 (Demirbas et al., 2001; Weng et al., 2001; Gao et al., 2005; Fan et al., 2009; Sugimoto et al., 2011, 2012; Sun et al., 2011; Wu et al., 2011; Lin et al., 2013; Zhang et al., 2013b; Li et al., 2016; Cheng et al., 2017; Niu et al., 2017; Zhong et al., 2017). The *Rps1* locus includes five alleles *Rps1a*, *Rps1b*, *Rps1c*, *Rps1d*, and *Rps1k* (Sugimoto et al., 2012). However, since the mapping intervals of these *Rps* genes on the short arm of chromosome 3 are close to or overlap with each other, it is difficult to confirm whether these genes are alleles or adjacent to each other. The individual *Rps* gene is non-durable due to the virulence complexity and rapid changes of *P. sojae* population. The emergence of new *P. sojae* pathotypes can quickly overcome the *Rps* genes (Dorrance et al., 2003a; Grau et al., 2004). Most of identified *Rps* genes would be effective for only 8–15 years under the pressures of emerging *P. sojae* pathotypes (Dorrance et al., 2004; Sugimoto et al., 2012; Stewart et al., 2014, 2016; Xue et al., 2015). No *Rps* gene that confers resistance to all *P. sojae* races or pathotypes in China has been identified (Zhu et al., 2003; Cui et al., 2010; Zhang et al., 2010, 2014). Among the *Rps* genes identified and mapped in China, some of them can effectively resist Chinese *P. sojae* pathotypes, like *RpsYD29*, *RpsYD25*, and *RpsQ* (Zhang et al., 2014; Li et al., 2017). Compared with *Rps1k*, *RpsYD25*, which was identified in the soybean cultivar Yudou25 in Henan province of China, showed a more broad-spectral resistance *P. sojae* pathotypes in China (Chen et al., 2008; Zhang et al., 2014; Li et al., 2017). Two independent studies have been carried out to map the *Rps* gene in Yudou25 (Fan et al., 2009; Sun et al., 2011). Although both studies have mapped *RpsYD25* on the short arm of chromosome 3, however, due to the different set of polymorphic molecular markers used in each mapping population and the relatively small size of mapping population, the linkage markers and their order in genetic maps in both studies are inconsistent. Thus, it is necessary for the larger-size mapping population and more polymorphic molecular markers to construct the high-resolution genetic and accurate physical maps for *RpsYD25*. This helps to confirm genomic intervals containing *RpsYD25*, identify candidate genes and further develop functional markers, which can be applied to marker-assisted breeding.

As the origin of soybean, there are abundant soybean germplasm resources, and a lot of germplasm resistant to *P. sojae* have been identified in recent decades in China (Zhu et al., 2003; Chen et al., 2008; Zhang et al., 2014). Although some studies have carried out the *Rps* gene postulation and prediction of Chinese soybean germplasm or cultivars based on resistance reaction types to different *P. sojae* isolates, due to the complex *P. sojae* pathotypes and genetic background of soybean germplasm, it is yet unclear about the distribution of identified *Rps* genes in the soybean cultivars in China (Chen and Wang, 2017). Fine mapping *Rps* genes and further development of co-segregated markers can detect *Rps* genes in soybean cultivars and germplasm to make more effective use of *P. sojae*-resistance sources. In our previous study, we used *P*. *sojae* isolate PsMC1 to conduct phenotypic screening on two mapping populations of 82 and 98 F_2:3_ families, respectively, and initially mapped *RpsYD25* on chromosome 3 (Fan et al., 2009). Hence, we finely mapped *RpsYD25* using a larger mapping population to determine the genomic region that contains *RpsYD25*. Then candidate genes were analyzed, and corresponding co-segregated markers were developed. In addition, the detection of *RpsYD25* in soybean cultivars and landraces from soybean producing regions in China was carried out using co-segregated markers.

## Materials and Methods

### Plant Materials and Mapping Population Development

A set of cultivars/lines each containing one known *Rps* gene was used as differential hosts for PRR phenotyping (Table 1). Two soybean cultivars Williams and Yudou21 were used as susceptible control (Zhang et al., 2014). All of these cultivars/lines were obtained from Institute of Crop Sciences, Chinese Academy of Agriculture Sciences.

**TABLE 1:** Phenotyping of 25 different soybean cultivars’ resistance reactions to 14 *Phytophthora sojae* isolates.

Cultivar/line (*Rps* gene)	*Phytophthora sojae* isolate^*a*^														No. of resistance to isolates
	PsRace1	PsRace3	PsRace4	PsRace5	PsUSAR2	Ps41-1	PsAH4	PsMC1	PsNKI	PsFJ2	PsFJ3	PsJS2	Ps6497	Ps7063	
Harlon (*Rps1a*)	S	S	S	S	R	S	R	S	R	S	S	S	R	S	4
Harosoy13XX (*Rps1b*)	R	R	R	R	S	S	S	S	S	S	S	S	S	R	5
Williams79 (*Rps1c*)	R	R	R	R	R	R	S	R	R	R	R	S	R	R	12
PI103091 (*Rps1d*)	R	S	S	R	R	S	S	S	S	S	S	S	S	S	3
Williams 82 (*Rps1k*)	R	R	R	R	R	R	S	S	R	R	S	S	R	R	10
L76-988 (*Rps2*)	R	R	R	R	S	S	S	S	S	S	S	S	S	S	4
L83-570 (*Rps3a*)	R	R	R	R	R	S	S	S	S	S	S	S	R	S	6
PRX146-36 (*Rps3b*)	R	R	S	R	R	S	S	S	S	S	S	S	R	R	6
PRX145-48 (*Rps3c*)	R	R	R	R	S	S	S	S	S	S	S	S	S	R	5
L85-2352 (*Rps4*)	R	R	R	R	R	S	S	S	S	S	S	S	R	S	6
L85-3059 (*Rps5*)	R	R	R	R	S	S	S	S	S	S	S	S	R	S	5
Harosoy62XX (*Rps6*)	R	R	R	R	R	S	R	S	S	S	S	S	R	S	7
Harosoy (*Rps7*)	R	R	R	S	R	S	S	S	S	S	S	S	S	S	4
PI399073 (*Rps8*)	R	R	R	R	R	S	R	S	S	S	S	S	R	S	7
Ludou4 (*Rps9*)	R	R	R	R	R	R	R	R	R	R	S	R	R	R	13
Wandou15 (*Rps10*)	R	R	R	R	R	R	R	R	R	S	R	S	R	S	11
Youbian30 (*RpsYB30*)	R	R	S	R	R	R	S	S	R	R	S	S	S	S	7
Yudou29 (*RpsYD29*)	R	R	R	R	R	R	S	R	R	R	R	S	R	R	12
Qichadou1 (*RpsQ*)	R	R	R	R	R	R	R	R	R	R	S	R	R	R	13
Huachun18(*RpsHC18*)	R	R	R	R	R	R	R	R	R	R	R	R	R	S	13
**Yudou25 (*RpsYD25*)**	**R**	**R**	**R**	**R**	**R**	**R**	**S**	**R**	**R**	**S**	**R**	**S**	**R**	**R**	**11**
Zaoshu18 (*RpsZS18*)	R	R	R	R	R	R	S	S	R	R	R	S	R	S	10
**Zheng92116**	**R**	**R**	**R**	**R**	**R**	**R**	**S**	**R**	**R**	**S**	**R**	**S**	**R**	**R**	**11**
Meng8206 (*RpsHN*)	R	S	S	R	S	S	S	S	S	S	S	S	S	S	2
Yudou21	S	S	S	S	S	S	S	S	S	S	S	S	S	S	0
Williams (*rps*)	S	S	S	S	S	S	S	S	S	S	S	S	S	S	0

*^a^PsRace1, PsRace3, PsRace4, PsRace5, Ps41-1, and PsNKI were isolated from Heilongjiang Province, Northeast of China; PsAH4 and PsMC1 were isolated from Anhui province, Huang-Huai Region of China; PsFJ2 and PsFJ3 were isolated from Fujian province in China; PsJS2 was isolated from Jiangsu province of China; PsUSAR2 was the strain of race2 from the United States with pathotype changed (Zhang et al., 2014). Ps6497 and Ps7063 were the standard strains from United States (Dou et al., 2010; Song et al., 2013). The bolded terms are the cultivars containing RpsYD25.*

A population of 165 F_2:3_ families derived from Zaoshu18 × Yudou25 was used as initial mapping for *RpsYD25*. The F_1_ hybrids were self-pollinated to produce F_2_ individuals. Each F_2_ individual was self-pollinated and the seeds were obtained as F_2:3_ Families. Fine mapping population consisting of 1127 families was obtained from segregating families based on phenotypic result of the F_2:3_ population crossed by Yudou25 and Zaoshu18 described by Fan et al. (2009) (Supplementary Figure S1). All the remaining seeds of segregating families identified for phenotype and genotype were planted in the field to produce the large F_3:4_ population. To determine positional relationship between *RpsYD25* and *Rps1*, allelic test was deployed to an F_2_ population consisting of 402 individuals derived from a cross Williams 82 (containing *Rps1k*) × Yudou25.

### PRR Phenotyping of the Soybean Cultivars and Mapping Populations

Fourteen *P. sojae* isolates were used for characterizing phenotype of each cultivars containing *Rps* genes. The set of *P. sojae* isolates was provided by Institute of Crop Sciences, Chinese Academy of Agricultural Sciences, Northeast Agricultural University, and Nanjing Agricultural University. All *P. sojae* isolates were isolated from infected soybean plants of different soybean production regions in China except for Ps7063. All *P. sojae* isolates were activated and transferred to the V_8_ juice agar medium for use. 15–20 seeds of each cultivars were planted in 1000 mL paper cups. Each of the soybean cultivars sowed for 14 cups in order to inoculate 14 different isolates, respectively. Inoculum preparation and the applied hypocotyl-inoculation technique were operated using the protocol described by Zhang et al. (2014).

Two *P. sojae* isolates PsMC1 (virulence formula: *Rps1a*, *1b*, *1d*, *1k*, *2*, *3a*, *3b*, *3c*, *4*, *5*, *6*, *7*, *8*, *YB30*, and *RpsZS18*) and Ps7063 (virulence formula: *Rps1a*, *1d*, *2*, *3a*, *4*, *5*, *6*, *7*, *8*, *10*, *YB30*, and *ZS18*) were used to evaluate resistance reaction of parental cultivars and populations. 20–25 seeds of each family in the mapping populations were planted in paper cups. For the 165 F_2:3_ families crossed by Zaoshu18 and Yudou25, PsMC1 and Ps7063 were used to identify the phenotypes. PsMC1 were used to evaluate resistance response of the 1127 derived F_3:4_ families from heterozygous families crossed by Zaoshu18 and Yudou25. 402 F_2_ individuals crossed by Williams 82 × Yudou25 were inoculated by the *P. sojae* isolate Ps7063 for the allelic test. The corresponding parents of all populations were also inoculated as control.

After 6 days of inoculation, the number of dead seedlings was recorded. Families with 0–20%, 80–100%, and 21–79% dead seedlings were considered homozygous resistant (R), susceptible (S), and segregating (Rs), respectively (Gordon et al., 2006; Lin et al., 2013; Zhang et al., 2013a, b; Ping et al., 2016).

### Simple Sequence Repeat (SSR) Analyses and Genetic Mapping

Equivalent amounts of leaf tissues from every seedlings of each family were mixed and stored at −80°C. Genomics DNA was extracted using the Plant Genomic DNA Kit (Tiangen, Beijing, China). Based on the previous mapping results of Fan et al. (2009) and Sun et al. (2011), SSR on the short arm of soybean chromosome 3 were selected to screen for polymorphism between parental cultivars (Song et al., 2010; Sugimoto et al., 2012; Zhang et al., 2013b). Screening and identification of SSR markers was based on previous studies (Zhong et al., 2017). The screened polymorphic SSR markers were genotyped for corresponding mapping population.

Linkage analysis was conducted with MAPMAKER/EXP version 3.0 (Lincoln et al., 1993). Kosambi mapping function was employed to calculate the Genetic distances (Kosambi, 1943). A log-likelihood threshold of 3.0 was used to determine the linkage groups. The genetic linkage map of the molecular markers linked to *RpsYD25* was prepared using MapDraw version 2.1 (Liu and Meng, 2003).

### Identifying and Genotyping Recombination Events and Candidate Gene Discovery

Whole genome re-sequencing was applied to the two parental cultivars Zaoshu18 and Yudou25. Genomic DNA of the two cultivars was isolated, respectively, to construct Illumina libraries and sequenced on an Illumina HiSeq^TM^ 4000 by Biomarker Technologies (Beijing, China) (Zhong et al., 2017). SNPs and InDels between the two parental cultivars were detected using the SNP analyses software GATK (McKenna et al., 2010). The main detection process: (1) for the results obtained by BWA (Li and Durbin, 2009), Picard’s mark duplicate tool was used to remove duplicates. (2) InDel realignment was deployed to correct the error of the alignment result caused by the insertion or deletion. (3) Base recalibration was conducted using GATK to correct the base quality value. (4) GATK was used for variant calling. (5) Strict filtering was performed on SNP and InDel: 2 SNP within 5 bp were filter out; SNPs located 5 bp upstream or downstream from InDel were filtered out; Two InDels with a distance of less than 10 bp were filtered out. Of all the obtained SNP and InDel, only homozygous sites were selected for further development of molecular marker.

According to the results of SSR genetic mapping, InDels in the *RpsYD25* genomic interval were identified for development of the PCR-based makers (Li et al., 2017), and SNPs in the mapping interval were obtained and used to develop PCR-based makers for the Tetra-Primer ARMS–PCR assay (Ye et al., 2001; Collins and Ke, 2012). Genomic sequence of Williams 82 corresponding to the mapping interval of *RpsYD25* was downloaded from SoyBase^1^, and then new simple sequence repeat (SSR) motifs were searched and developed to SSR makers using the website primer design tool BatchPrimer3^2^ (You et al., 2008). Recombinant break points in the derived 1127 F_3:4_ families were identified using the developed makers in the genomic region containing *RpsYD25*. Gene models in the final genomic interval that no recombinant events occurred among the 1127 F_2:3_ families were preferred as candidate genes for *RpsYD25*.

### Co-segregating Marker Genotyping Among Soybean Cultivars

Soybean cultivars containing single *Rps* genes were genotyped by the identified markers co-segregated with *RpsYD25* to detect whether they can effectively distinguish other published *Rps* genes. Cluster analysis was deployed according to the polymorphic co-segregated markers in different soybean genotypes. Each marker polymorphic information content (PIC) was calculated among these genotypes using the online tool PICcalc (Nagy et al., 2012). A Neighbor-joining tree was constructed using the PowerMarker V3.0 and MEGA 6.0 software among the soybean genotypes, and further distinguish whether these markers can effectively identify the haplotype of *RpsYD25* (Liu and Muse, 2004; Tamura et al., 2013). To further detect *RpsYD25* in Chinese soybeans, 178 cultivars and landraces were selected to identify the reaction type to eight *P. sojae* isolates (PsRace1, PsRace3, PsRace4, PsRace5, PsUSAR2, Ps41-1, PsMC1, and PsJS2). The combination of co-segregated markers was also used to detect *RpsYD25* haplotype among these soybean genotypes.

## Results

### Phenotyping for Phytophthora Resistance

To identify the resistance reaction of *RpsYD25* and other known *Rps* genes, 25 soybean cultivars containing single known *Rps* genes along with Yudou25 were selected and inoculated with 14 *P. sojae* isolates to identify reaction types. The results revealed that the 25 soybean cultivars produced a total of 18 reaction types when inoculated the 14 isolates (Table 1). Among them, Williams and Yudou21 showed susceptibility to 14 isolates, confirming that the two cultivars do not contain *Rps* genes (Zhang et al., 2014). Yudou25 was resistant to 11 of 14 isolates, and the reaction type was different from all the other soybean genotypes containing known *Rps* genes except Zheng92116, suggesting that *RpsYD25* is a distinct *Rps* gene (Fan et al., 2009). Zheng92116 is a soybean cultivar derived from Yudou25, and Sun et al. (2011) showed that Zheng92116 contained the *RpsYD25* (*RpsYu25*).

Further analysis the reaction types between resistant cultivars Zaoshu18 and Yudou25, there is a difference in the response to three *P. sojae* isolates, namely PsMC1, PsFJ2 and Ps7063. Yudou25 showed resistance to PsMC1 and Ps7063, while Zaoshu18 were susceptible to both of them; Zaoshu18 was resistant to PsFJ2, while Yudou25 showed susceptibility to PsFJ2. Therefore, in order to identify the resistance gene *RpsYD25* in Yudou25, isolates PsMC1 and Ps7063 were selected for phenotypic evaluation of 165 F_2:3_ families derived from Yudou25 and Zaoshu18 (Table 1). The phenotyping results of the 165 F_2:3_ families inoculated with isolates PsMC1 and Ps7063 were consistent. Among F_2:3_ families, 37 were homozygous resistant families, 82 were segregating families, and 46 were susceptible families. The χ^2^ test revealed that phenotyping results fit well with 1: 2: 1 ratio (χ^2^ = 0.99 < χ^2^_0__.__05_ = 5.99) (Table 2 and Supplementary Figures S2A,B). Among the 1127 families of the F_3:4_ sub-population derived from the cross between Zaoshu18 and Yudou25, there were 281 homozygous resistant, 544 segregated, and 302 susceptible. The χ^2^ test met the 1:2:1 ratio (χ^2^ = 2.13 < χ^2^_0.05_ = 5.99) (Table 2 and Supplementary Figure S2C). Therefore, it indicates that Phytophthora resistance in Yudou25 is controlled by a single dominant gene, which is consistent with previous studies (Fan et al., 2009; Sun et al., 2011).

**TABLE 2:** Reactions of F_2_ individuals and F_2:3_ families derived from crosses between parental soybean cultivars to *Phytophthora sojae* isolates.

*P. sojae* isolates	Parent and the cross	Generation	Amount	Observed number^*a*^			Chi squared tests		
				R^*a*^	Rs	S	Expected ratio	χ*^2^*	*P*
PsMC1, Ps7063	Zaoshu18 × Yudou25	F_2:3_	165	37	82	46	1:2:1	0.99	0.61
PsMC1	Zaoshu18 × Yudou25	F_3:4_	1127	281	544	302	1:2:1	2.13	0.34
Ps7063	Williams 82 × Yudou25	F_2_	402	402		0			

*^*a*^R, homozygous resistant; Rs, segregating; S, susceptible.*

402 F_2_ plants from the cross between Williams 82 and Yudou25 were identified for phenotypes using isolate Ps7063. All 402 individuals showed resistance and no plants died. Allele test indicated that *RpsYD25* on chromosome 3 may be an allele of or closely linked with *Rps1k* (Table 2).

### Genetic Map Construction for *RpsYD25*

**FIGURE 1:**
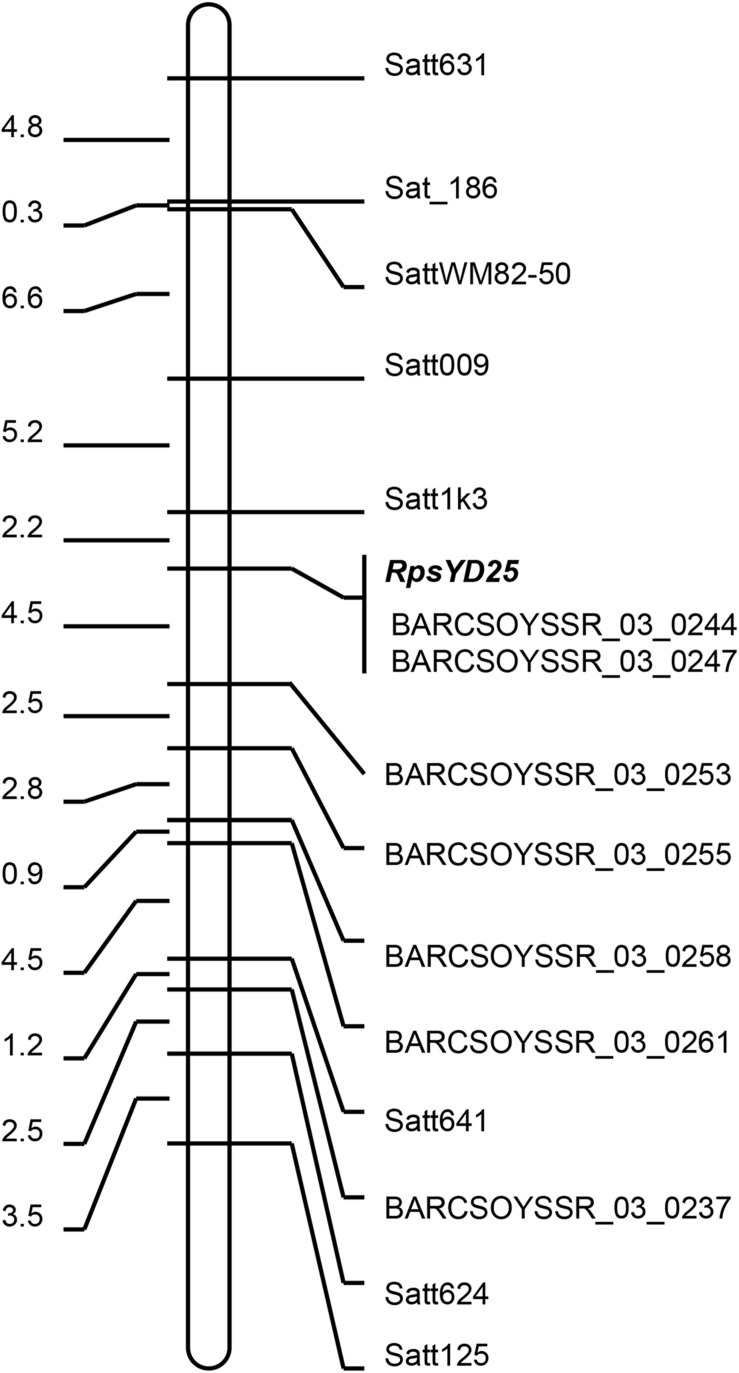
Genetic linkage map of *RpsYD25* on chromosome 3. The genetic distances (cM) are on the left and the molecular markers are on the right.

Based on the mapping results of *RpsYD25* by Fan et al. (2009) and Sun et al. (2011), 120 SSR markers which had been published on the short arm of chromosome 3 were selected to screen polymorphism between Zaoshu18 and Yudou25. 15 SSR markers showed polymorphism between the two parental cultivars. The SSR markers were further used to identify the genotypes of 165 F_2:3_ families, and the linkage map of *RpsYD25* was constructed based on the phenotype and genotype results. *RpsYD25* was mapped between SSR markers Satt1k3 and BARCSoysSR_03_0253 with genetic distances of 2.2 cM (LOD = 71.68, 86.5% variation explained) and 4.5 cM (LOD = 60.37, 81.5% variation explained), respectively, while BARCSOYSSR_03_0244 and BARCSOYSSR_03_0247 were co-segregated with *RpsYD25* (Figure 1).

### Fine Mapping of *RpsYD25*

**FIGURE 2:**
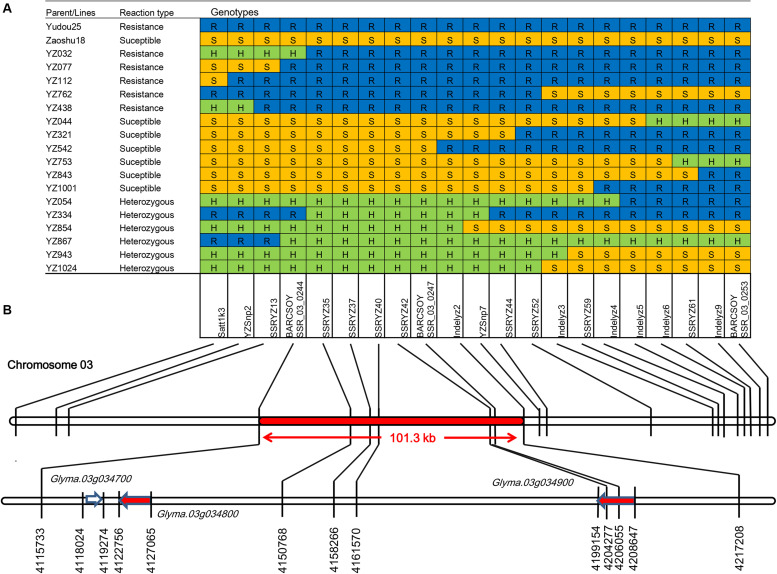
Fine mapping of the *RpsYD25* locus through the identification of recombinants carrying crossovers. **(A)** Recombinants were identified by genotyping recombinant F_3:4_ families using developed molecular makers. *Phytophthora sojae* isolate PsMC1 was used for phenotypic identification of the mapping population. Blue, yellow, and green grids represent resistant, susceptible and heterozygous segments, respectively, as determined by molecular markers. **(B)** Physical positions of molecular markers and gene models (according to the Williams 82 reference genome, Glyma.Wm82.a2). The red arrows indicate the NBS-LRR genes.

Based on the preliminary mapping results of *RpsYD25*, *RpsYD25* was located between SSR marker Satt1k3 and BARCSOYSSR_03_0253. The primer sequences of Satt1k3 were searched on Williams 82 (*Glycine max* version 2.0) reference genome, and Satt1k3 was located at 4022513 bp ∼ 4022856 bp on chromosome 3, while BARCSOYSSR_03_0253 is located at 4132642 bp ∼ 4132683 bp on chromosome 3. The physical distance between the two SSR markers is approximately 290 kb. Identification of recombinant families was carried out in 1127 F_3:4_ families derived from Zaoshu18 and Yudou25 using SSR markers Satt1k3 and BARCSOYSSR_03_0253. On the Satt1k3 side, a total of 6 recombination events were identified; On the BARCSOYSSR_03_0253 side, 12 recombination events were occurred in 12 different families (Figure 2A).

In order to further narrow the mapping interval of *RpsYD25*, we carried out the detection of further recombination sites in 17 recombinant families by developing different types of molecular markers in the *RpsYD25*-mapped region (Figure 2A). PCR markers were developed based on SNP and InDel sites identified within the *RpsYD25* genomic interval identified by whole genome re-sequencing. After detecting amplification efficiency and polymorphism between Zaoshu18 and Yudou25, two Tetra-AMS PCR-based polymorphic SNP markers YZSnp2 and YZSnp7 (Supplementary Table S1) and six polymorphic InDel markers Indelyz2, Indelyz3, Indelyz4, Indelyz5, Indelyz6, and Indelyz9 were identified (Supplementary Table S2). Based on the physical interval of the Willimas82 genome sequence (*Glycine max* V2.0), new SSR loci were searched and developed as SSR markers. Nine new polymorphic SSR markers showed polymorphism between parental cultivars (Supplementary Table S3).

The newly developed two SNP markers, six InDel markers, and nine SSR markers and two co-segregated SSR markers from the genetic mapping were used to further identify the recombination sites among 17 recombinant families. The results show that BARCSOYSSR_03_0244 has the closest recombination event from *RpsYD25* in families YZ334 and YZ032, and on the other side, the closest recombination event happened at Indelyz2 in family YZ542 (Figure 2A). The distance between the BARCSOYSSR_03_0244 and Indelyz2 is 101.3 kb, and between them there are five co-segregated SSR markers, namely SSRYZ35, SSRYZ37, SSRYZ40, SSRYZ42, and BARCSOYSSR_03_0247. There are three gene models in physical region of *RpsYD25*, Glyma.03g034700 is a gene model with zinc ion binding and nucleic acid binding protein structure (Zinc ion binding; nucleic; acid binding), Glyma.03g034800 and Glyma.03g034900 are typical disease-resistant structure of the NBS-LRR gene. These three genes may be candidate gene models of *RpYD25* (Figure 2B).

### Validation of Co-segregated Markers for Specific Detection of *RpsYD25*

**FIGURE 3:**
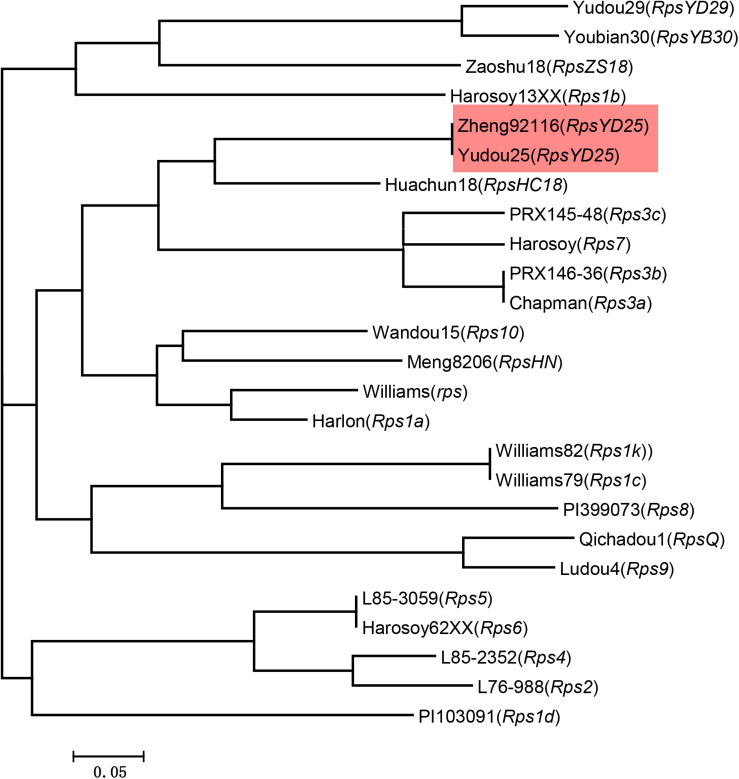
Dendrogram of the 25 soybean cultivars reflected by Neighbor-joining cluster analysis based on molecular band of five *RpsYD25* co-segregated SSR markers using the softwares Powermarker and Mega 6.0.

To detect whether the co-segregated markers can effectively distinguish *RpsYD25* haplotype from other identified *Rps* genes, these markers were used to detect soybean genotypes containing known *Rps* genes and susceptible control genotypes. Among the 25 soybean genotypes, the PIC values of the five co-segregated markers ranged from 0.62 to 0.84, which means that these markers are relatively rich in polymorphism among different genotypes (Table 3). Cluster analysis showed that the five markers formed 21 haplotypes in 25 soybean genotypes (Figure 3). Yudou25 and Zheng92116 belonged to the same haplotype, indicating that both Zheng92116 and Yudou25 contained *RpsYD25*.

**TABLE 3:** Co-segregating SSR markers in the *RpsYD25* mapping interval and the polymorphic information contents (PICs) among 25 different soybean cultivars.

Marker name		Reverse primer	SSR motif	Start site	Stop site	Product (bp)	Polymorphic information content (PIC)
SSRYZ35	Forward primer	ACGGTCATCTGATTATAAATTG	(AT)24	4150768	4150894	127	0.80
	Reverse primer	AGTGTGAAATAGTGTGCGTGT					
SSRYZ37	Forward primer	CATTATTTTGTCCGCCTATAA	(AG)11	4158266	4158412	147	0.62
	Reverse primer	TATATCAAGGTTTGGACGTGT					
SSRYZ40	Forward primer	ATTCCGTCACTAAACTGCATA	(TA)9	4161570	4161692	123	0.84
	Reverse primer	TAAACATAAAGCGTGACAACA					
SSRYZ42	Forward primer	ATGACACATGCTAATTGATCC	(TA)6	4204277	4204469	192	0.78
	Reverse primer	CGCCATTTCAAAAGAATTAC					
BARCSOY	Forward primer	ATTATTATGGTGGGGCGTGA	(TAA)17	4205985	4206148	163	0.83
SSR_03_0247	Reverse primer	TGACCACCATTTCAAAGGAA					

**FIGURE 4:**
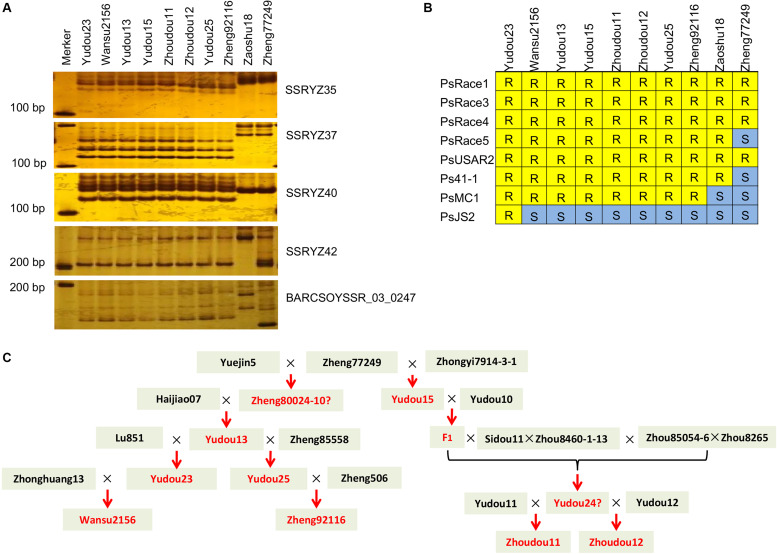
*RpsYD25* haplotype detection among 178 soybean genotypes. **(A)** Soybean genotypes with the same molecular band types as “Yudou25” and “Zheng92116.” **(B)** Resistance reaction types to eight *Phytophthora sojae* isolates of soybean genotypes containing the *RpsYD25* haplotype and soybean genotypes without *RpsYD25*, “Zaoshu18” or “Williams 82.” **(C)** Analysis of pedigree relationships among soybean genotypes containing the *RpsYD25* haplotype. Red indicates soybean genotypes containing *RpsYD25.* The soybean genotype with “?” cannot be molecularly detected owing to the failure to obtain materials.

Molecular marker detection using the five co-segregated markers was performed on 178 soybean genotypes. Six genotypes with the same haplotype as Yudou25 and Zheng92116 were identified, namely Yudou23, Wansu2156, Yudou13, Yudou15, Zhoudou11, and Zhoudou12 (Figure 4A). The resistance reaction of the seven soybean cultivars to eight *P. sojae* isolates was consistent with that of Yudou25 except Yudou23, which showed resistant all the eight *P. sojae* isolates (Figure 4B and Supplementary Table S4), this may be due to an allelic mutation or other resistance genes existed in Yudou23. Pedigree analysis showed that these cultivars are genetically related to Yudou25 (Figure 4C). The seven cultivars all have the same ancestral cultivar Zheng77249, but Zheng77249 is different in molecular band and resistance reaction type from the derivatives. For phenotypic identification, Zheng77249 has a phenotypic difference with Yudou25 on three isolates (PsRace5, Ps41-1 and PsMC1), and for molecular identification, Zheng77249 is the same as Yudou25 only at the SSR marker YZSSR42 locus (Figures 4A,B and Supplementary Table S4), suggesting that Zheng77249 does not contain *RpsYD25* haplotype. These results show that the combination of the five co-segregated markers can effectively identify soybean genotypes containing *RpsYD25* haplotype.

## Discussion

*RpsYD25* can effectively resist the *P. sojae* pathotypes in China. Zhang et al. (2014) analyzed the resistance spectrum of soybean cultivars in Henan Province, and found that Yudou25 were resistant to 21 of the 26 *P. sojae* isolates; Li et al. (2017) inoculated Yudou25 with 36 different *P. sojae* isolates in different areas of China, and found that Yudou25 showed resistance to 33 of 36 isolates. In this study, Yudou25 was inoculated with 14 different virulence isolates of *P. sojae*, and it showed resistant to 11 of them. In the comparison of the resistance reactions of 25 soybean genotypes to 14 *P. sojae* isolates, the reaction type of Yudou25 was only the same as that of Zheng92116, and it is different from other soybean genotypes containing *Rps* genes, which indicates that Zheng92116 may contain *RpsYD25*. Zheng92116 is a soybean cultivar from the hybrid of Yudou25 as the male parent and Zheng506 as the female parent. Sun et al. (2011) found that the *Rps* gene in Zheng92116 is likely to be the same as Yudou25 through genetic mapping and pedigree analysis, and this result was also verified by *RpsYD25* co-segregated SSR markers detection in this study.

At present, there are 15 *Rps* genes which are located on the short arm of chromosome 3, however, many *Rps* genes cannot be determined the positional relationship between each other, because the mapping intervals were usually too large or the order of markers cannot correspond to the reference genome (Demirbas et al., 2001; Weng et al., 2001; Gao et al., 2005; Fan et al., 2009; Schmutz et al., 2010; Sugimoto et al., 2011, 2012; Sun et al., 2011; Wu et al., 2011; Lin et al., 2013; Zhang et al., 2013b; Li et al., 2016; Cheng et al., 2017; Niu et al., 2017; Zhong et al., 2017). Previous studies have mapped *RpsYD25* in a large region of chromosome 3 (Fan et al., 2009; Sun et al., 2011). Therefore, in this study, we first reconstructed the genetic linkage map of *RpsYD25* with the published SSR markers on chromosome 3, confirming the previous mapping results, and narrowed the mapping interval to a region of 290 kb. To further finely map *RpsYD25*, we reconstructed a sub-population containing 1127 F_3:4_ families using the resistant segregated families selected from the F_2:3_ populations derived by the cross of Zaoshu18 and Yudou25 constructed by Fan et al. (2009). The nearly flanking markers of *RpsYD25* were used to identify the recombinant families and recombination sites in the population, and the mapping interval of *RpsYD25* was further reduced to 101.3 kb.

**FIGURE 5:**
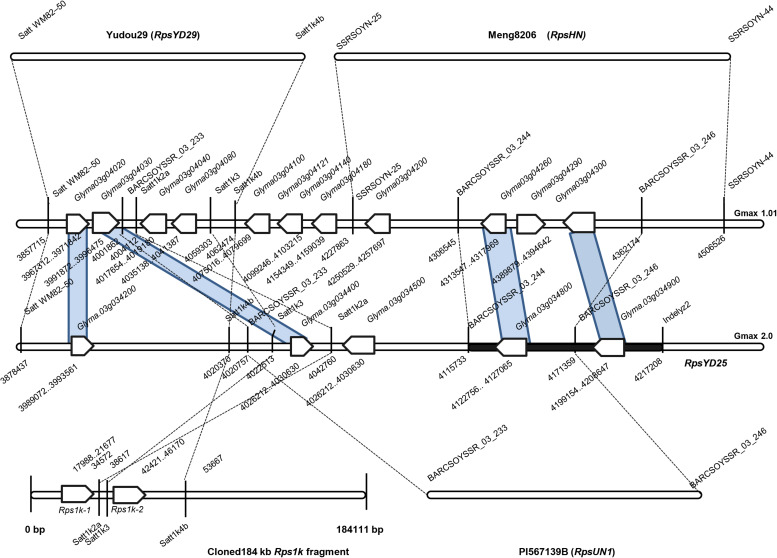
Integration physical map containing *Rps* genes which were mapped near *RpsYD25* genomic region on soybean chromosome 3. The white arrow represents genes with the NBS-LRR structure in the region.

In the mapping interval of *RpsYD25*, there are two resistance gene models with NBS-LRR structure, Glyma.03g034800 and Glyma.03g034900, and a gene Glyma.03g034700 with zinc ion and nucleic acid binding domain structure. The mapping interval of *RpsYD25* is partially overlapped with the *Rps* genes *RpsUN1* and *RpsHN* (Figure 5). The candidate gene Glyma.03g034800 is also predicted to be a candidate for *RpsUN1* (Li et al., 2016), while Glyma.03g034800 and Glyma.03g034900 are located within the *RpsHN* mapping region and are predicted to be candidate genes for *RpsHN* (Niu et al., 2017). In this study, we performed a comparative analysis of the resistance patterns of soybean cultivar Meng8206 containing *RpsHN* and Yudou25, and found that Meng8206 was only resistant to 2 isolates PsRace1 and PsRace5, while Yudou25 is resistant to 11 isolates. Niu et al. (2017) used 8 *P. sojae* isolates to identify the resistance spectrum of Meng8206, and the results showed that Meng8206 was only resistant to one isolate. Because *RpsYD25* and *RpsHN* showed significant difference in the resistance types to *P. sojae*, these two genes are unlikely to be the same *Rps* gene, presumably alleles or tightly linked genes. Haplotype analysis also indicates that the two cultivars contain different *Rps* genes (Figure 3). *RpsUN1* was identified in US soybean germplasm PI 567139B. PI 567139B is from Indonesia. Due to lack of this resource, we were unable to perform resistance reaction type analysis and molecular marker identification in this study. The relationship between *RpsYD25* and *RpsUN1* is currently uncertain. Although *Rps1k* has been cloned, no identical sequence to the cloned *Rps1k* gene sequence was found in the Williams 82 genome sequence, making it difficult to determine the exact locus of *Rps1k* in the reference genome. In this study, we performed an allelic test on the F_2_ population derived from the hybridization of Williams 82 and Yudou25. It was found that *RpsYD25* and *Rps1k* did not separate in 402 individuals, which proved that the two genes are alleles or tightly linked. The SSR marker Satt1k3, which is closest to the genetic distance of *RpsYD25*, is from the *Rps1k* clone sequence (Zhang et al., 2013b), so it is speculated that *RpsYD25* is closely linked to *Rps1k* (Figure 5).

Although zinc ion binding gene was not used as an *Rps* candidate gene in previous studies (Li et al., 2016; Niu et al., 2017), barley powdery mildew resistance gene *Rar1* containing zinc ion binding domain was able to increase the accumulation of H_2_O_2_ through signal transduction, triggered by race-specific resistance (R) genes (Shirasu et al., 1999). Therefore, we speculate that the gene *Glyma.03g034700* may be triggered by the race-specific NBS-LRR genes *Glyma.03g034800* and *Glyma.03g034900* in soybean to resist *P. sojae*. *RpsYD25* is mapped in a genomic region rich in NBS-LRR gene clusters on chromosome 3. Among the identified resistance genes, *RpsYD29*, *RpsUN1*, and *RpsHN* were mapped in the region containing the NBS-LRR gene cluster, and all located in the 200 kb region upstream and downstream of *RpsYD25* (Figure 5; Zhang et al., 2013b; Li et al., 2016; Niu et al., 2017). *Rps1k* has been cloned to prove that consist of two adjacent NBS-LRR genes. This study also confirmed that *Rps1k* should be located near the *RpsYD25* region by allelic test and molecular mapping analysis (Gao et al., 2005; Gao and Bhattacharyya, 2008). The NBS-LRR genes are typical resistance genes expressing conserved domains of the nucleotide-binding site and leucine-rich repeats. Among the resistance genes in plants that have been cloned and characterized, 61% of them belong to NBS-LRR gene (Shao et al., 2016; Kourelis and van der Hoorn, 2018; Xing et al., 2018). The accumulation of mutations in the NBS-LRR gene can increase the complexity of the NBS-LRR gene clusters and can generate new resistance types by recombining or rearranging with each other, such as gene duplication, unequal exchange, chromosomal abnormal recombination, gene conversion (Michelmore and Meyers, 1998; Meyers et al., 2003; Nagy and Bennetzen, 2008; Marone et al., 2013; Lestari et al., 2017; Lv et al., 2017). The resistance mechanism in the NBS-LRR gene cluster region is complicated. In the cloning and functional study of the wheat powdery mildew resistance gene *Pm60*, it was found that the NBS-LRR powdery mildew resistance gene can interact with its adjacent NBS-LRR structural gene to jointly provide resistance against powdery mildew (Zou et al., 2018). The diversity of *Rps* genes on soybean chromosome 3 may be caused by the translocation, recombination or rearrangement of functional NBS-LRR genes in the NBS-LRR gene cluster to lead the different resistance types to *P. sojae* (Kang et al., 2012; Lestari et al., 2017). It requires further experimental support to explore whether there is a large sequence change in the mapping interval of *RpsYD25* compared with the reference genome Williams82 such as the presence of other genes.

Due to the large number of high similarity NBS-LRR sequences around *RpsYD25* region, it is difficult to effectively detect specific mutation sites in candidate genes based on whole genome re-sequencing, and to develop specific primers for amplifying target candidate genes. However, in the mapping interval of *RpsYD25*, five SSR markers with high PIC were found to effectively distinguish the haplotype of *RpsYD25* from other known *Rps* genes. Therefore, these SSR markers can be used to jointly detect *RpsYD25* haplotype in different soybean genotypes. The *RpsYD25* haplotype was detected in 8 related soybean genotypes, which all have broad-spectrum resistance to *P. sojae* in China (Zhang et al., 2014). However, the resistance types of Yudou23 was not the same as that of other genotypes in this study, and 7 other genotypes were consistent to the resistance reaction to 8 *P. sojae* isolates, but they differ in their reactions to individual *P. sojae* isolates in the study of Zhang et al. (2014), which may be due to virulence changes of the *P. sojae* isolates or other alleles existed in *RpsYD25* locus. The *RpsYD25* absence in the shared ancestor genotypes Zheng77249 may be due to the unequal recombination in the *RpsYD25* locus in the process of deriving other genotypes, resulting in stronger resistance to *P. sojae*, which may also explain why the Henan province in China is not the area where PRR severely occurs, but there is evidence for abundance resources containing resistance diversity to *P. sojae* (Zhang et al., 2014). Hence, Zheng77249 in this study may not be the original material used to produce Yudou25 and Yudou15, and have a novel *RpsYD25* allele generated after multiple generations.

In the present study, the Phytophthora resistance gene *RpsYD25* was finely mapped, and three candidate genes for *RpsYD25* were identified in the mapping interval. *RpsYD25* was found to be located in a genomic region rich in NBS-LRR genes. The co-segregated markers with *RpsYD25* was developed and identified, and can be further converted into high-throughput markers such as SNP or KASP to improve the efficiency of marker-assisted selection to detect the *RpsYD25* haplotype.

## Data Availability Statement

The datasets generated for this study can be found in the NCBI: [SAMN15376405] [https://www.ncbi.nlm.nih.gov/biosample/15376405], [SAMN15376406][https://www.ncbi.nlm.nih.gov/biosample/15376406], [SAMN15376407][https://www.ncbi.nlm.nih.gov/biosample/15376407], and [SAMN15376408][https://www.ncbi.nlm.nih.gov/biosample/15376408].

## Author Contributions

ZZ conceived and designed the experiments and revised the manuscript. CZ, SS, XZ, and CD performed the experiments. CZ analyzed the data and wrote the manuscript. All authors read and approved the manuscript.

## Conflict of Interest

The authors declare that the research was conducted in the absence of any commercial or financial relationships that could be construed as a potential conflict of interest.

## Footnotes

1 https://www.soybase.org/

2 https://wheat.pw.usda.gov/demos/BatchPrimer3/

## References

[B1] Chen X. Wang Y. (2017). “Phytophthora sojae,” in *‘Biological invasions and its management in China’*, eds Wan F. Jiang M. Zhan A. (Singapore: Springer), 199–223.

[B2] Chen X. Zhu Z. Wang X. Xiao Y. Wu X. (2008). Postulation of Phytophthora resistance genes in soybean cultivars or lines. *Sci. Agric. Sin.* 41 1227–1234.

[B3] Cheng Y. Ma Q. Ren H. Xia Q. Song E. Tan Z. (2017). Fine mapping of a Phytophthora-resistance gene *RpsWY* in soybean (*Glycine max* L.) by high-throughput genome-wide sequencing. *Theor. Appl. Genet.* 130 1041–1051. 10.1007/s00122-017-2869-5 28246754 PMC5395582

[B4] Collins A. Ke X. (2012). Primer1: primer design web service for tetra-primer ARMS-PCR. *Open Bioinform. J.* 6 55–58. 10.2174/1875036201206010055

[B5] Cui L. Yin W. Tang Q. Dong S. Zheng X. Zhang Z. (2010). Distribution, pathotypes, and metalaxyl sensitivity of *Phytophthora sojae* from Heilongjiang and Fujian provinces in China. *Plant Dis.* 94 881–884. 10.1094/PDIS-94-7-0881 30743553

[B6] Demirbas A. Rector B. G. Lohnes D. G. Fioritto R. J. Graef G. L. Cregand P. B. (2001). Simple sequence repeat markers linked to the soybean genes for Phytophthora resistance. *Crop Sci.* 41 1220–1227. 10.2135/cropsci2001.4141220x

[B7] Dorrance A. E. (2018). Management of *Phytophthora sojae* of soybean: a review and future perspectives. *Can. J. Plant Pathol.* 40 210–219. 10.1080/07060661.2018.1445127

[B8] Dorrance A. E. Jia H. Abney T. S. (2004). Evaluation of soybean differentials for their interaction with *Phytophthora sojae*. *Plant Health Prog.* 5:9. 10.1094/PHP-2004-0309-01-RS

[B9] Dorrance A. E. McClure S. A. DeSilva A. (2003a). Pathogenic diversity of *Phytophthora sojae* in Ohio soybean fields. *Plant Dis.* 87 139–146. 10.1094/PDIS.2003.87.2.139 30812918

[B10] Dorrance A. E. McClure S. A. St. Martin S. K. (2003b). Effect of partial resistance on Phytophthora stem rot incidence and yield of soybean in Ohio. *Plant Dis.* 87 308–312. 10.1094/PDIS.2003.87.3.308 30812766

[B11] Dou D. Kale S. D. Liu T. Tang Q. Wang X. Arredondo F. D. (2010). Different domains of *Phytophthora sojae* effector Avr4/6 are recognized by soybean resistance genes Rps4 and Rps6. *Mol. Plant Microbe* 23 425–435. 10.1094/MPMI-23-4-0425 20192830

[B12] Fan A. Wang X. Fang X. Wu X. Zhu Z. (2009). Molecular identification of Phytophthora resistance gene in soybean cultivar Yudou 25. *Acta Agron. Sin.* 35 1844–1850. 10.3724/sp.j.1006.2009.01844

[B13] Gao H. Bhattacharyya M. K. (2008). The soybean-Phytophthora resistance locus *Rps1-k* encompasses coiled coil-nucleotide binding leucine rich repeat-like genes and repetitive sequences. *BMC Plant Biol.* 8:29. 10.1186/1471-2229-8-29 18366691 PMC2330051

[B14] Gao H. Narayanan N. N. Ellison L. Bhattacharyya M. K. (2005). Two classes of highly similar coiled coil-nucleotide binding-leucine rich repeat genes isolated from the Rps1-k locus encode Phytophthora resistance in soybean. *Mol. Plant Mcrobe* 18 1035–1045. 10.1094/MPMI-18-1035 16255242

[B15] Gordon S. G. St. Martin S. K. Dorrance A. E. (2006). *Rps8* maps to a resistance gene rich region on soybean molecular linkage group F. *Crop Sci.* 46 168–173. 10.2135/cropsci2004.04-0024

[B16] Grau C. R. Dorrance A. E. Bond J. Russin J. S. (2004). “Fungal diseases,” in *Soybeans: Improvement, Production, and Uses, (Soybeans Improve)*, ed. Wilcox J.R. (Madison, WI: American Society of Agronomy), 679–763.

[B17] Kamoun S. Furzer O. Jones J. D. Judelson H. S. Ali G. S. Dalio R. J. (2015). The Top 10 oomycete pathogens in molecular plant pathology. *Mol. Plant Pathol.* 16 413–434. 10.1111/mpp.12190 25178392 PMC6638381

[B18] Kang Y. J. Kim K. H. Shim S. (2012). Genome-wide mapping of *NBS-LRR* genes and their association with disease resistance in soybean. *BMC Plant Biol.* 12:139. 10.1186/1471-2229-12-139 22877146 PMC3493331

[B19] Kaufmann M. Gerdemann J. (1958). Root and stem rot of soybean caused by *Phytophthora sojae* n. sp. *Phytopathology* 48 201–208.

[B20] Koenning S. R. Wrather J. A. (2010). Suppression of soybean yield potential in the continental United States by plant diseases from 2006 to 2009. *Plant Health Prog.* 11:5. 10.1094/PHP-2010-1122-01-RS

[B21] Kosambi D. D. (1943). The estimation of map distances from recombination values. *Ann. Eugen.* 12 172–175. 10.1007/978-81-322-3676-4-16

[B22] Kourelis J. van der Hoorn R. A. L. (2018). Defended to the nines: 25 years of resistance gene cloning identifies nine mechanisms for R protein function. *The Plant Cell* 30, 285–299. 10.1105/tpc.17.00579 29382771 PMC5868693

[B23] Lestari P. Lee S. H. Tasma I. M. (2017). Gene Duplication to reveal adaptation clue of plant to environmental stress: a case study of *NBS-LRR* genes in soybean. *J. Agro. Biogen.* 12 119–130.

[B24] Li H. Durbin R. (2009). Fast and accurate short read alignment with Burrows–Wheeler transform. *Bioinformatics* 25 1754–1760. 10.1093/bioinformatics/btp324 19451168 PMC2705234

[B25] Li L. Lin F. Wang W. Ping J. Fitzgerald J. Zhao M. (2016). Fine mapping and candidate gene analysis of two loci conferring resistance to *Phytophthora sojae* in soybean. *Theor. Appl. Genet.* 129 2379–2386. 10.1007/s00122-016-2777-0 27591777

[B26] Li Y. Sun S. Zhong C. Wang X. Wu X. Zhu Z. (2017). Genetic mapping and development of co-segregating markers of *RpsQ*, which provides resistance to *Phytophthora sojae* in soybean. *Theor. Appl. Genet.* 130 1223–1233. 10.1007/s00122-017-2883-7 28258371

[B27] Lin F. Zhao M. Ping J. Johnson A. Zhang B. Abney T. S. (2013). Molecular mapping of two genes conferring resistance to *Phytophthora sojae* in a soybean landrace PI 567139B. *Theor. Appl. Genet.* 126 2177–2185. 10.1007/s00122-013-2127-4 23689748

[B28] Lincoln S. E. Daly M. J. Lander E. S. (1993). *Constructing Genetic Maps with MAPMAKER/EXP Version 3.0: A Tutorial and Reference Manual*, 3rd Edn Cambridge: Whitehead Institute for Biomedical Research.

[B29] Liu K. Muse S. (2004). *PowerMarker: A New Genetic Data Analysis Software. Version 3.0.*

[B30] Liu R. H. Meng J. L. (2003). MapDraw: a microsoft excel macro for drawing genetic linkage maps based on given genetic linkage data. *Hereditas* 25 317–321. 15639879

[B31] Lv Q. Huang Z. Xu X. Tang L. Liu H. Wang C. (2017). Allelic variation of the rice blast resistance gene *Pid3* in cultivated rice worldwide. *Sci. Rep.* 7:10362. 10.1038/s41598-017-10617-2 28871108 PMC5583387

[B32] Marone D. Russo M. A. Laidò G. De Leonardis A. M. Mastrangelo A. M. (2013). Plant nucleotide binding site-leucine-rich repeat (NBS-LRR) genes: active guardians in host defense responses. *Int. J. Mol. Sci.* 14 7302–7326. 10.3390/ijms14047302 23549266 PMC3645687

[B33] McKenna A. Hanna M. Banks E. Sivachenko A. Cibulskis K. Kernytsky A. (2010). The Genome Analysis Toolkit: a MapReduce framework for analyzing next-generation DNA sequencing data. *Genome Res.* 20, 1297–1303. 10.1101/gr.107524.110 20644199 PMC2928508

[B34] Meyers B. C. Kozik A. Griego A. Kuang H. Michelmore R. W. (2003). Genome-wide analysis of NBS-LRR–encoding genes in *Arabidopsis*. *Plant Cell* 15 809–834. 10.1105/tpc.009308 12671079 PMC152331

[B35] Michelmore R. W. Meyers B. C. (1998). Clusters of resistance genes in plants evolve by divergent selection and a birth-and-death process. *Genome Res.* 8 1113–1130. 10.1101/gr.8.11.1113 9847076

[B36] Nagy E. D. Bennetzen J. L. (2008). Pathogen corruption and site-directed recombination at a plant disease resistance gene cluster. *Genome Res.* 18 1918–1923. 10.1101/gr.078766.108 18719093 PMC2593579

[B37] Nagy S. Poczai P. Cernák I. Gorji A. M. Hegedûs G. Taller J. (2012). PICcalc: an online program to calculate polymorphic information content for molecular genetic studies. *Biochemical. Genet.* 50 670–672. 10.1007/s10528-012-9509-1 22573137

[B38] Niu J. Guo N. Sun J. Li L. Cao Y. Li S. (2017). Fine mapping of a resistance gene *RpsHN* that controls *Phytophthora sojae* using recombinant inbred lines and secondary populations. *Front. Plant Sci.* 8:538. 10.3389/fpls.2017.00538 28443124 PMC5387331

[B39] Ping J. Fitzgerald J. C. Zhang C. Lin F. Bai Y. Wang D. (2016). Identification and molecular mapping of *Rps11*, a novel gene conferring resistance to *Phytophthora sojae* in soybean. *Theor. Appl. Genet.* 129 445–451. 10.1007/s00122-015-2638-2 26660465

[B40] Sahoo D. K. Abeysekara N. S. Cianzio S. R. Robertson A. E. Bhattacharyya M. K. (2017). A Novel *Phytophthora sojae* resistance *Rps12* gene mapped to a genomic region that contains several Rps genes. *PLoS One* 12:e0169950. 10.1371/journal.pone.0169950 28081566 PMC5233422

[B41] Schmitthenner A. F. (1985). Problems and progress in control of Phytophthora root rot of soybean. *Plant Dis.* 69 362–368.

[B42] Schmitthenner A. F. (1999). “Phytophthora rot of soybean,” in *Compendium of Soybean Diseases*, 4th Edn, eds Hartman G. L. Rupe J. C. Sikora E. J. Domier L. L. Davis J. A. Steffey K. L. (St. Paul, Minnesota: The American Phytopathological Society Press), 39–42.

[B43] Schmutz J. Cannon S. B. Schlueter J. Ma J. Mitros T. Nelson W. (2010). Genome sequence of the palaeopolyploid soybean. *Nature* 463 178–183. 10.1038/nature08670 20075913

[B44] Shao Z. Q. Xue J. Y. Wu P. Zhang Y. M. Wu Y. Hang Y. Y. (2016). Large-scale analyses of angiosperm nucleotide-binding site-leucine-rich repeat (NBS-LRR) genes reveal three anciently diverged classes with distinct evolutionary patterns. *Plant Physiol.* 170 2095–2109. 10.1104/pp.15.01487 26839128 PMC4825152

[B45] Shirasu K. Lahaye T. Tan M. W. Zhou F. Azevedo C. Schulze-Lefert P. (1999). A novel class of eukaryotic zinc-binding proteins is required for disease resistance signaling in barley and development in *C. elegans*. *Cell* 99 355–366. 10.1016/S0092-8674(00)81522-6 10571178

[B46] Song Q. Jia G. Zhu Y. Grant D. Nelson R. T. Hwang E. Y. (2010). Abundance of SSR motifs and development of candidate polymorphic SSR markers (BARCSOYSSR_1.0) in soybean. *Crop Sci.* 50 1950–1960. 10.2135/cropsci2009.10.0607

[B47] Song T. Kale S. D. Arredondo F. D. Shen D. Su L. Liu L. (2013). Two RxLR avirulence genes in *Phytophthora sojae* determine soybean *Rps* 1k-mediated disease resistance. *Mol. Plant Microbe Interact.* 26, 711–720. 10.1094/MPMI-12-12-0289-R 23530601

[B48] Stewart S. Abeysekara N. Robertson A. E. (2014). Pathotype and genetic shifts in a population of *Phytophthora sojae* under soybean cultivar rotation. *Plant Dis.* 98 614–624. 10.1094/PDIS-05-13-0575-RE 30708552

[B49] Stewart S. Robertson A. E. Wickramasinghe D. Draper M. A. Michel A. Dorrance A. E. (2016). Population structure among and within Iowa, Missouri, Ohio, and South Dakota populations of *Phytophthora sojae*. *Plant Dis.* 100 367–379. 10.1094/PDIS-04-15-0437-RE 30694137

[B50] Sugimoto T. Kato M. Yoshida S. Matsumoto I. Kobayashi T. Kaga A. (2012). Pathogenic diversity of *Phytophthora sojae* and breeding strategies to develop Phytophthora-resistant soybeans. *Breed. Sci.* 61 511–522. 10.1270/jsbbs.61.511 23136490 PMC3406798

[B51] Sugimoto T. Yoshida S. Kaga A. Hajika M. Watanabe K. Aino M. (2011). Genetic analysis and identification of DNA markers linked to a novel *Phytophthora sojae* resistance gene in the Japanese soybean cultivar Waseshiroge. *Euphytica* 182 133–145. 10.1007/s10681-011-0525-8

[B52] Sun J. Li L. Zhao J. Huang J. Yan Q. Xing H. (2014). Genetic analysis and fine mapping of *RpsJS*, a novel resistance gene to *Phytophthora sojae* in soybean [*Glycine max* (L.)Merr.]. *Theor. Appl. Genet.* 127 913–919. 10.1007/s00122-014-2266-2 24419901

[B53] Sun S. Wu X. Zhao J. Wang Y. Tang Q. Yu D. (2011). Characterization and mapping of *RpsYu25*, a novel resistance gene to *Phytophthora sojae*. *Plant Breed.* 130 139–143. 10.1111/j.1439-0523.2010.01794.x

[B54] Tamura K. Stecher G. Peterson D. Filipski A. Kumar S. (2013). MEGA6: molecular evolutionary genetics analysis version 6.0. *Mol. Biol. Evol.* 30 2725–2729. 10.1093/molbev/mst197 24132122 PMC3840312

[B55] Tian M. Zhao L. Li S. Huang J. Sui Z. Wen J. (2016). Pathotypes and metalaxyl sensitivity of *Phytophthora sojae* and their distribution in Heilongjiang, China 2011-2015. *J. Gen. Plant Pathol.* 82 132–141. 10.1007/s1032

[B56] Tooley P. W. Grau C. R. (1984). The relationship between rate-reducing resistance to *Phytophthora megasperma* f. sp. glycinea and yield of soybean. *Phytopathology* 74 1209–1216.

[B57] Tyler B. M. (2007). *Phytophthora sojae*: root rot pathogen of soybean and model oomycete. *Mol. Plant Pathol.* 8 1–8. 10.1111/j.1364-3703.2006.00373.x 20507474

[B58] Weng C. Yu K. Anderson T. R. Poysa V. (2001). Mapping genes conferring resistance to Phytophthora root rot of soybean, *Rps1a* and *Rps7*. *J. Hered.* 92 442–446. 10.1093/jhered/92.5.442 11773256

[B59] Wu M. Li B. Liu P. Weng Q. Zhan J. Chen Q. (2016). Population genetic analyses of *Phytophthora sojae* in Fujian, China. *Plant Pathol.* 66 1182–1190. 10.1111/ppa.12666

[B60] Wu X. Zhang B. Sun S. Zhao J. Yang F. Guo N. (2011). Identification, genetic analysis and mapping of resistance to *Phytophthora sojae* of *Pm28* in soybean. *Agric. Sci. China* 10 1506–1511. 10.1016/S1671-2927(11)60145-4

[B61] Xing L. Hu P. Liu J. Witek K. Zhou S. Xu J. (2018). *Pm21* from *Haynaldia villosa* encodes a CC-NBS-LRR that confers powdery mildew resistance in wheat. *Mol. Plant* 11 874–878. 10.1016/j.molp.2018.02.013 29567451

[B62] Xue A. G. Marchand G. Chen Y. Zhang S. Cober E. R. Tenuta A. (2015). Races of *Phytophthora sojae* in Ontario, Canada, 2010-2012. *Can. J. Plant Pathol.* 37 376–383. 10.1080/07060661.2015.1052562

[B63] Ye S. Dhillon S. Ke X. Collins A. R. Day I. N. (2001). An efficient procedure for genotyping single nucleotide polymorphisms. *Nucleic Acids Res.* 29:e88. 10.1093/nar/29.17.e88 11522844 PMC55900

[B64] You F. M. Huo N. Gu Y. Q. Luo M. C. Ma Y. Hane D. (2008). BatchPrimer3: a high throughput web application for PCR and sequencing primer design. *BMC Bioinformatics* 9:253. 10.1186/1471-2105-9-253 18510760 PMC2438325

[B65] Zhang J. Sun S. Wang G. Duan C. Wang X. Wu X. (2014). Characterization of Phytophthora resistance in soybean cultivars/lines bred in Henan province. *Euphytica* 196 375–384. 10.1007/s10681-013-1040-x

[B66] Zhang J. Xia C. Duan C. Sun S. Wang X. Wu X. (2013a). Identification and candidate gene analysis of a novel Phytophthora resistance gene Rps10 in a Chinese soybean cultivar. *PLoS One* 8:e69799. 10.1371/journal.pone.0069799 23936102 PMC3723638

[B67] Zhang J. Xia C. Wang X. Duan C. Sun S. Wu X. (2013b). Genetic characterization and fine mapping of the novel Phytophthora resistance gene in a Chinese soybean cultivar. *Theor. Appl. Genet.* 126 1555–1561. 10.1007/s00122-013-2073-1 23467992

[B68] Zhang S. Xu P. Wu J. Zhang J. Li W. Chen C. (2010). Races of *Phytophthora sojae* and their Virulences on soybean cultivars in Heilongjiang, China. *Plant Dis.* 94 87–91. 10.1094/PDIS-94-1-0087 30754393

[B69] Zhong C. Sun S. Li Y. Duan C. Zhu Z. (2017). Next-generation sequencing to identify candidate genes and develop diagnostic markers for a novel Phytophthora resistance gene, RpsHC18, in soybean. *Theor. Appl. Genet.* 131 525–538. 10.1007/s00122-017-3016-z 29138903

[B70] Zhong C. Sun S. Yao L. Ding J. Duan C. Zhu Z. (2018). Fine mapping and identification of a novel phytophthora root rot resistance locus RpsZS18 on Chromosome 2 in Soybean. *Front. Plant Sci.* 9:44. 10.3389/fpls.2018.00044 29441079 PMC5797622

[B71] Zhu Z. Wang H. Wang X. Chang R. Wu X. (2003). Distribution and virulence diversity of *Phytophthora sojae* in China. *Agric. Sci. China* 3 116–123.

[B72] Zou S. Wang H. Li Y. Kong Z. Tang D. (2018). The NB-LRR gene *Pm60* confers powdery mildew resistance in wheat. *New Phytol.* 218 298–309. 10.1111/nph.14964 29281751

