# Supplementary material for: Fine Mapping, Candidate Gene Identification and Co-segregating Marker Development for the Phytophthora Root Rot Resistance Gene RpsYD25
Source: Front Genet. 2020 Jul 28;11:799. doi: 10.3389/fgene.2020.00799 (PMC7399351; doi:10.3389/fgene.2020.00799)
Supplement: Supplementary file 1 [file Table_1.docx]

**Table S1** Primers for SNPs based on Tetra-AMRS PCR

| Name | Position | Reference genome | Yudou25 | Zaoshu18 | Primer sequence | |
| --- | --- | --- | --- | --- | --- | --- |
| YZSnp2 | 4038036 | T | A | T | Forward inner primer (A allele) | TCATATATTTTTTGGTTTAATGAGGGT |
|  |  |  |  |  | Reverse inner primer (C allele) | AATAAAATGGTGATCAAATCTTCAGTT |
|  |  |  |  |  | Forward outer primer (5’ – 3’) | CCTGATAGTTTGTACCTTAGAAAGTGAG |
|  |  |  |  |  | Reverse outer primer (5’ – 3’) | ACTTCTCCTAACTCTATTTCAGAAATGG |
| YZSnp7 | 4223193 | C | A | C | Forward inner primer (T allele): | CCTTTAGACCTCTTAAATTGACGATCGA |
|  |  |  |  |  | Reverse inner primer (A allele): | ATTTTACGGTAGCAACACATGATACGTG |
|  |  |  |  |  | Forward outer primer (5’ – 3’): | TCATTTGAAAATCCATTTTTTTGGATTC |
|  |  |  |  |  | Reverse outer primer (5’ – 3’): | ATCAATTGTATCATCAAAAGCAGTCCAA |
