# Supplementary material for: Fine Mapping, Candidate Gene Identification and Co-segregating Marker Development for the Phytophthora Root Rot Resistance Gene RpsYD25
Source: Front Genet. 2020 Jul 28;11:799. doi: 10.3389/fgene.2020.00799 (PMC7399351; doi:10.3389/fgene.2020.00799)
Supplement: Supplementary file 2 [file Table_2.docx]

| InDel name | Position | Yudou25 | Zaoshu18 |  | Primer sequence (5'-3') |
| --- | --- | --- | --- | --- | --- |
| Indelyz2 | 4217208 | G | GCTT | Forward primer | TGCATATCATTGCAGCAACTT |
|  |  |  |  | Reverse primer | TACCCCTACACGACCAGCAT |
| Indelyz3 | 4290741 | TATTTAGTA | T | Forward primer | GAAGTGTAGTCCTCCAACTCCA |
|  |  |  |  | Reverse primer | AGTTGGTTCCCTAACCCCCA |
| Indelyz4 | 4294103 | AATGGGTTCAGGTAT | A | Forward primer | TCGGTGGTTCAATTCTACGGT |
|  |  |  |  | Reverse primer | CAACCGACCTTTCCTACCCA |
| Indelyz5 | 4301666 | CTTAACTTCTA | C | Forward primer | CATCTACTGCAGTTACAAAGTTACA |
|  |  |  |  | Reverse primer | CAGCATGGGTTTTGCATCTCC |
| Indelyz6 | 4301768 | AAACCCATGCC | A | Forward primer | GCTTTCTGAAGGCAAAACAATTT |
|  |  |  |  | Reverse primer | GCAAGACACAGACAAATCAACTCA |
| Indelyz9 | 4308271 | GCCCTCGATCATCA | G | Forward primer | TCGTACAAGAGGAGAGGGGC |
|  |  |  |  | Reverse primer | AAAGTCACGGACGCAGGAAA |

**Table S2** InDels used for developing PCR markers
