# Supplementary material for: Fine Mapping, Candidate Gene Identification and Co-segregating Marker Development for the Phytophthora Root Rot Resistance Gene RpsYD25
Source: Front Genet. 2020 Jul 28;11:799. doi: 10.3389/fgene.2020.00799 (PMC7399351; doi:10.3389/fgene.2020.00799)
Supplement: Supplementary file 3 [file Table_3.docx]

**Table S3** SSR marker primer sequences searched and developed based on reference genome

| Marker name |  | Reverse primer | SSR motif | Start site | Stop site | Product（bp） |
| --- | --- | --- | --- | --- | --- | --- |
| SSRYZ13 | Forward primer | CAAATGGTTACATGCACAAA | (AT)6 | 4042814 | 4043032 | 219 |
|  | Reverse primer | GTCCTGCTATTCTGATCCTCT |  |  |  |  |
| SSRYZ35 | Forward primer | ACGGTCATCTGATTATAAATTG | (AT)24 | 4150768 | 4150894 | 127 |
|  | Reverse primer | AGTGTGAAATAGTGTGCGTGT |  |  |  |  |
| SSRYZ37 | Forward primer | CATTATTTTGTCCGCCTATAA | (AG)11 | 4158266 | 4158412 | 147 |
|  | Reverse primer | TATATCAAGGTTTGGACGTGT |  |  |  |  |
| SSRYZ40 | Forward primer | ATTCCGTCACTAAACTGCATA | (TA)9 | 4161570 | 4161692 | 123 |
|  | Reverse primer | TAAACATAAAGCGTGACAACA |  |  |  |  |
| SSRYZ42 | Forward primer | ATGACACATGCTAATTGATCC | (TA)6 | 4204277 | 4204469 | 193 |
|  | Reverse primer | CGCCATTTCAAAAGAATTAC |  |  |  |  |
| SSRYZ44 | Forward primer | AACTGATAGCTACTCGGGAAA | (TATT)4 | 4226008 | 4226158 | 151 |
|  | Reverse primer | GAACCTTTTACCCATTTGATT |  |  |  |  |
| SSRYZ52 | Forward primer | GTTTGTGTCTGTGACTCAGTG | (AT)6 | 4265949 | 4266095 | 147 |
|  | Reverse primer | AAGAGACAACCACAAACACAC |  |  |  |  |
| SSRYZ59 | Forward primer | GAGATGCTTCCAACTAAGTCA | (AT)27 | 4291750 | 4291897 | 148 |
|  | Reverse primer | CTATTTTTAGTTTGAGGTTCTGA |  |  |  |  |
| SSRYZ61 | Forward primer | CATTTTTCAAGGCCAGTTAAT | (TTA)5 | 4307605 | 4307856 | 252 |
|  | Reverse primer | TGCTTAAAGGTTTTACTGCAA |  |  |  |  |
