# Supplementary material for: Fine Mapping, Candidate Gene Identification and Co-segregating Marker Development for the Phytophthora Root Rot Resistance Gene RpsYD25
Source: Front Genet. 2020 Jul 28;11:799. doi: 10.3389/fgene.2020.00799 (PMC7399351; doi:10.3389/fgene.2020.00799)
Supplement: Supplementary file 5 [file Data_Sheet_1.docx]

Supplementary Material

Fine mapping, candidate gene identification and co-segregating marker development for the Phytophthora root rot resistance gene *RpsYD25* in soybean

Chao Zhong^1, 2^, Suli Sun^1^, Canxing Duan^1^, and Zhendong Zhu^1^

^1^National Key Facility for Crop Gene Resources and Genetic Improvement, Institute of Crop Sciences, Chinese Academy of Agricultural Sciences, Beijing, 100081, China

^2^College of Agronomy, Shenyang Agricultural University, Shenyang, China

**^*^Corresponding author:** Zhendong Zhu

E-mail: zhuzhendong@caas.cn

Tel.: +86-10-82109609; Fax: +86-10-82109608

# Supplementary Tables

**
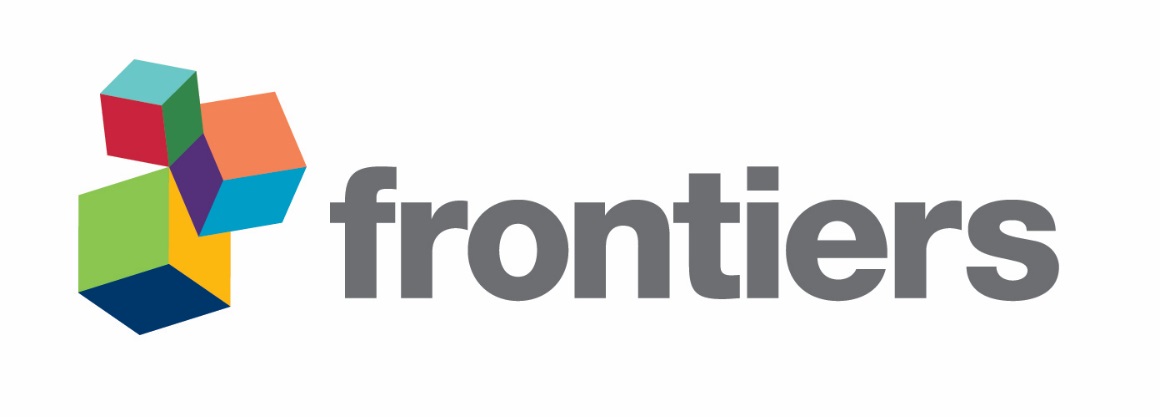
**

**Table S1** Primers for SNPs based on Tetra-AMRS PCR

**Table S2** InDels used for developing PCR markers

**Table S3** SSR marker primer sequences searched and developed based on reference genome

**Table S4** Reaction types of 178 soybean genotypes after inoculation with 8 *P. sojea* isolates

**Figure S1** Schematic representation of the construction of derived F_3:4_ population.

**Figure S2** Frequency distribution of proportion of dead individuals in mapping populations. **(A)** Frequency distribution of proportion of dead individuals in F_2:3_ families inoculated with *P. sojae* isolate PsMC1. **(B)** Frequency distribution of proportion of dead individuals in F_2:3_ families inoculated with *P. sojae* isolate Ps7063. **(C)** Frequency distribution of proportion of dead individuals in F_3:4_ families inoculated with *P. sojae* isolate PsMC1.
