# Supplementary figures and images for: Fine Mapping, Candidate Gene Identification and Co-segregating Marker Development for the Phytophthora Root Rot Resistance Gene RpsYD25
Source: Front Genet. 2020 Jul 28;11:799. doi: 10.3389/fgene.2020.00799 (PMC7399351; doi:10.3389/fgene.2020.00799)

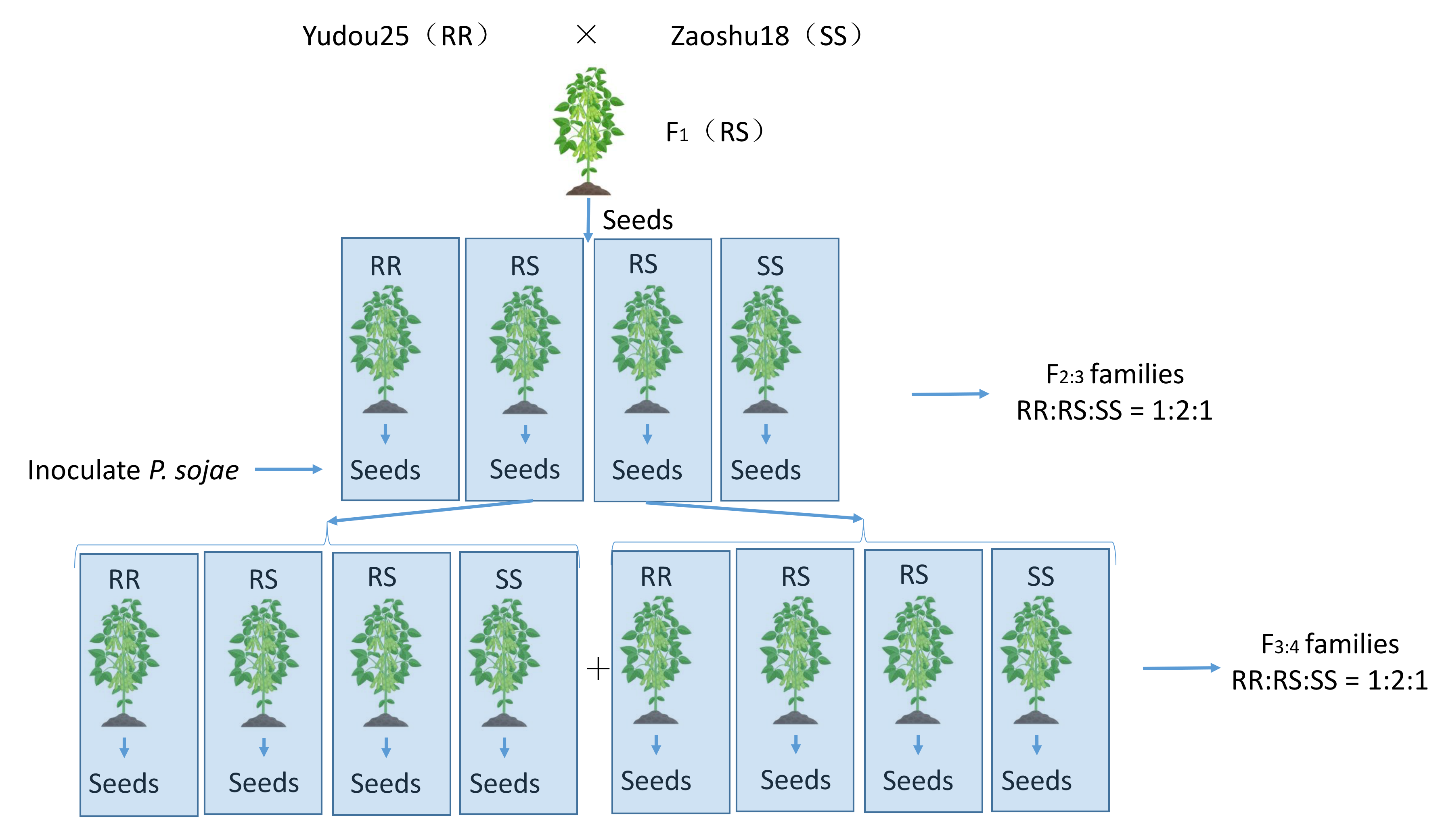

Supplement: Supplementary file 6 [file Image_1.TIF]

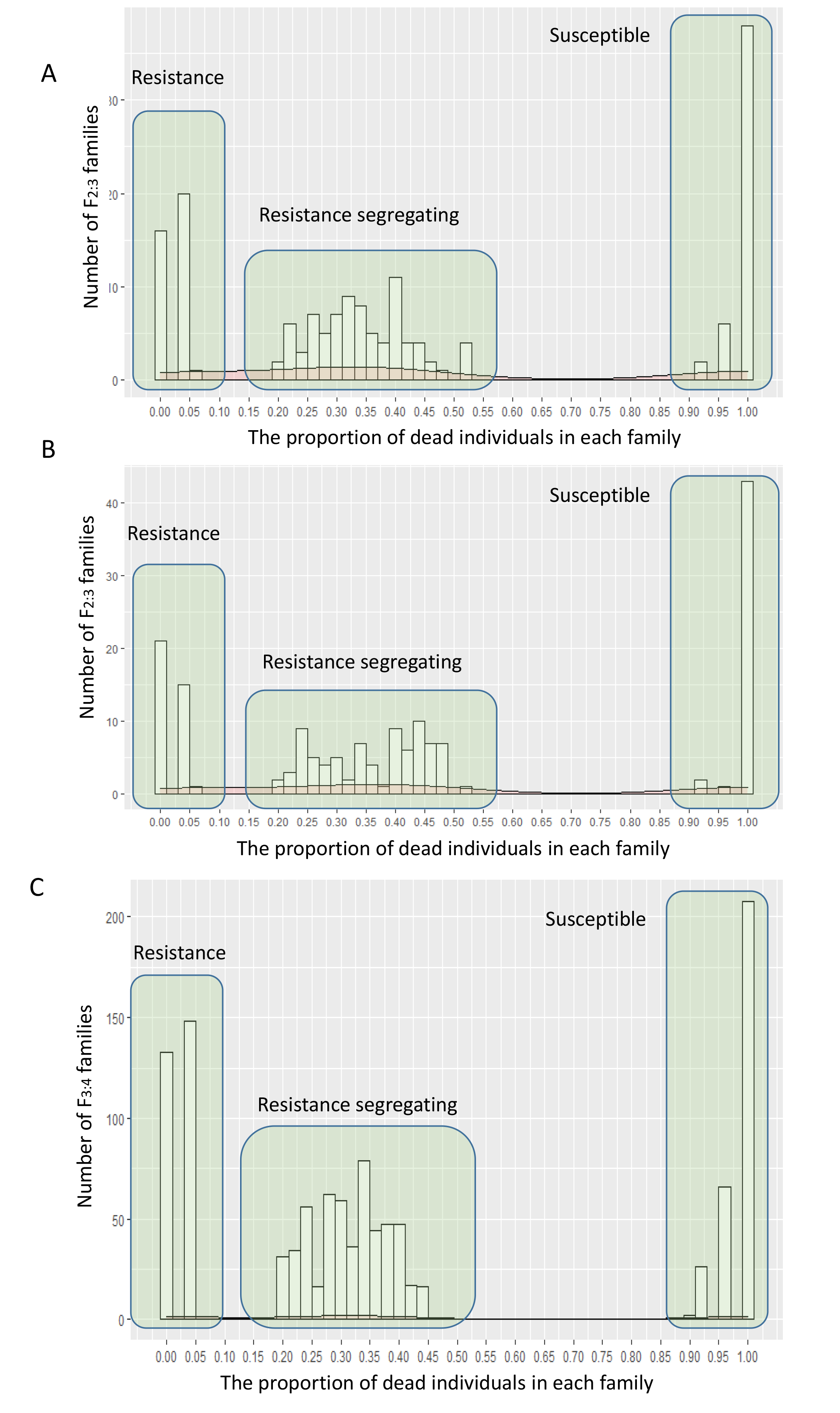

Supplement: Supplementary file 7 [file Image_2.TIF]
